# The Paradox of Physical Activity and Coronary Artery Calcification: Implications for Cardiovascular Risk

**DOI:** 10.3390/jcm13216523

**Published:** 2024-10-30

**Authors:** Da-Eun Sung, Ki-Chul Sung

**Affiliations:** 1 Department of Internal Medicine, Samsung Medical Center, Sungkyunkwan University School of Medicine, Seoul 06351, Republic of Korea; eunnyjoy@gmail.com; 2 Division of Cardiology, Department of Internal Medicine, Kangbuk Samsung Hospital, Sungkyunkwan University School of Medicine, Saemunan-ro, Jongno-gu, Seoul 03181, Republic of Korea

**Keywords:** coronary artery calcification, physical activity, cardiovascular health, atherosclerosis, plaque stability

## Abstract

The introduction of CT scans and the subsequent Agatston score in the 1990s drastically improved our ability to detect coronary artery calcification (CAC). This led to its incorporation into cardiovascular risk assessment guidelines set forth by organizations such as the American Heart Association (AHA) and the American College of Cardiology (ACC). Over time, these guidelines have evolved significantly, reflecting an increasing understanding of CAC. Physical activity has become a key factor in the management of cardiovascular disease. However, the relationship between physical activity and CAC remains complex. Although physical activity is generally beneficial for cardiovascular health, paradoxically, high levels of physical activity have been associated with elevated CAC scores. However, these higher CAC levels may indicate the presence of more stable, calcified plaques that provide protection against plaque rupture. These contradictory findings call for balanced interpretations that acknowledge the cardiovascular benefits of physical activity. This review examines the historical development of clinical guidelines for CAC, the paradoxical relationship between physical activity and CAC, and potential underlying mechanisms. It emphasizes the need for future research to utilize objective measures and consistent methodologies to better understand the relationship between physical activity and CAC.

## 1. Introduction

Coronary artery calcification (CAC) has long been recognized as a key marker in atherosclerosis research and is a strong predictor of cardiovascular events [1,2]. Quantifying CAC via computed tomography (CT) scans, typically reported as an Agatston score, offers a non-invasive measure of calcified plaque burden in coronary arteries [3]. Higher CAC scores are consistently linked to increased risks of adverse cardiovascular outcomes, underscoring their clinical utility in risk stratification. Additionally, arterial stiffness is associated with both arteriosclerosis [4] and diabetes [5], and is closely linked to CAC [6], further emphasizing its broader implications for cardiovascular health.

Physical activity (PA) is universally acknowledged as essential for cardiovascular health, offering a range of benefits, including enhanced cardiorespiratory fitness, improved lipid metabolism, better blood pressure regulation, optimized glucose homeostasis, and reduced systemic inflammation [7,8,9]. These physiological adaptations collectively contribute to PA’s protective effect against cardiovascular diseases (CVDs). As a result, authoritative organizations consistently recommend regular PA as a cornerstone of a healthy lifestyle [10].

The association between CAC and traditional cardiovascular risk factors, such as higher lipid values, hypertension, and arterial stiffness, has been well-documented [11,12,13,14,15,16,17,18,19,20,21,22,23,24]. However, the relationship between PA and CAC is less clear. Emerging evidence suggests that high levels of PA, particularly vigorous-intensity PA, might be associated with increased CAC scores. This finding is counterintuitive given the well-established cardiovascular benefits of exercise. This paradox, where PA both protects against CVD and potentially increases CAC, calls for a deeper understanding of the underlying mechanisms and clinical implications.

This review aims to comprehensively examine the historical development of clinical guidelines for CAC, as well as the paradoxical relationship between PA and CAC. We will explore the interplay between PA and CAC, highlighting both supportive and contradictory evidence. By synthesizing current research findings, we aim to reconcile these seemingly contradictory observations and provide a nuanced understanding of the underlying mechanisms. 

## 2. Historical Perspective and Evolution of Guidelines on Coronary Artery Calcification

Studies on calcification date back centuries, with evidence of calcified arteries even found in ancient mummies, indicating that atherosclerosis has affected humans for millennia [25]. Early autopsy studies identified calcified plaques within coronary arteries, establishing a clear link between calcification and atherosclerosis [26]. This foundational knowledge has driven further research and technological advancements.

The development of computed tomography (CT) scans revolutionized the detection and quantification of CAC. The introduction of the Agatston score in the 1990s provided a standardized measure of calcification, significantly enhancing cardiovascular risk assessment [27]. The development of non-invasive imaging technologies, such as electron beam CT and multi-detector CT, has enabled the precise visualization and quantification of coronary calcification, facilitating large-scale studies and improving risk stratification. As a result, major professional organizations, including the American Heart Association (AHA), the American College of Cardiology (ACC), and the European Society of Cardiology (ESC), have incorporated CAC scoring into their cardiovascular risk assessment guidelines. 

The role of CAC scoring in clinical guidelines has evolved significantly since its initial introduction. Initially proposed in the 2000 ACC/AHA guidelines as a potential tool for assessing CAD risk, it was not until 2007 that CAC scoring received a formal Class IIa recommendation. By 2010, the ACC/AHA guidelines upheld this recommendation, emphasizing the role of CAC scoring alongside traditional risk factors in intermediate-risk adults and older patients with diabetes, though its universal use was not advocated. However, in 2013, the ACC/AHA guidelines downgraded CAC scoring to a Class IIb recommendation due to concerns about radiation exposure, cost, and limited data. By 2018, CAC scoring was recommended for refining risk assessments, with a CAC score of 0 indicating no need for statin therapy unless other conditions were present, and a CAC score of ≥100 supporting statin initiation [28]. The 2024 ESC guidelines place greater emphasis on the importance of CAC [29]. For individuals with suspected chronic coronary syndrome and a low pre-test likelihood of obstructive CAD (5–15%), CAC scoring is now recommended to reclassify risk and identify those with a very low (≤5%) CACS-weighted clinical likelihood. In asymptomatic individuals with prior chest CT scans showing coronary artery calcification, these findings should be used to improve risk stratification and guide the management of modifiable risk factors.

Globally, guidelines are increasingly recognizing the value of CAC scoring in cardiovascular risk assessment [30]. The Australia and New Zealand guidelines (CSANZ) recommend CAC scoring for risk assessment and therapy decisions as follows: withholding statins when CAC = 0, favoring lifestyle changes when CAC = 1–100, recommending statins when CAC = 101–400 if above the 75th percentile, and necessitating statin therapy when CAC > 400 [31]. The Canadian Cardiovascular Society (CCS) guidelines primarily advocate for the use of CAC scoring for intermediate-risk patients (FRS 10–19.9%) to refine risk stratification and suggest repeating CAC scoring every five years if statins are withheld [32]. In East Asian countries, although CAC may be considered less critical than in Western nations, its importance is increasingly recognized. Japanese guidelines recommend CAC as a prognostic tool for intermediate- to high-risk individuals, though they call for additional studies due to Japan’s lower CAD morbidity and mortality rates compared to Western populations [33]. Chinese guidelines appreciate CAC scoring for aspirin allocation, although they primarily focus on traditional risk factors, such as blood pressure, cholesterol, smoking status, and age, for cardiovascular risk assessment [34].

## 3. Physical Activity and CAC

As CAC scoring becomes increasingly central in clinical guidelines, understanding its relationship with PA, especially concerning cardiovascular health, has garnered significant attention. Recent studies highlight a paradoxical association between high levels of PA and increased CAC scores. This section reviews key studies that have reported on the complex relationship between PA and CAC.

### 3.1. Physical Activity and Increased CAC Scores

Multiple studies have demonstrated an association between high PA levels and elevated CAC scores. In a 25-year follow-up study of 3175 participants from the CARDIA study, Laddu et al. found that individuals with PA levels exceeding recommended guidelines had a higher likelihood of having a CAC score above zero [35]. This association was particularly pronounced in White males who engaged in high levels of PA, with increased odds of developing detectable CAC (AOR 1.86, 95% CI: 1.16–2.98). These findings suggest that high PA levels may increase CAC risk in certain demographic groups.

**Figure 1:**
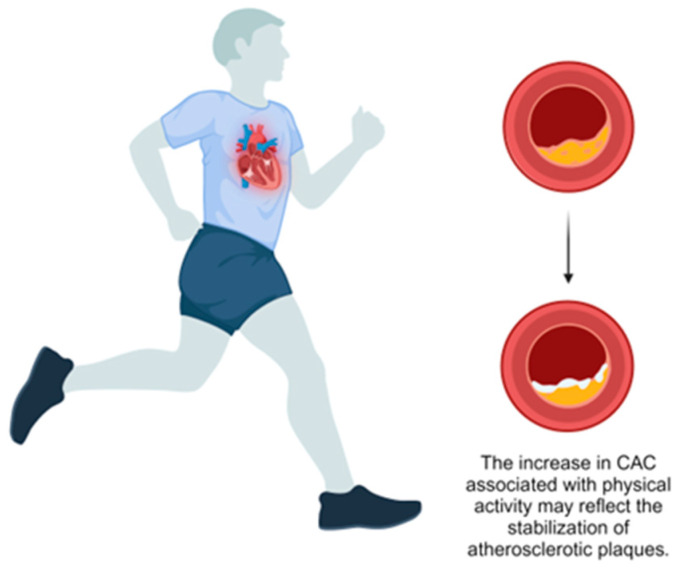
Effect of physical activity on coronary artery calcification.

Supporting this, Sung et al. conducted a large prospective study of 25,485 healthy adults and found that individuals classified as health-enhancing physically active (HEPA) had higher baseline CAC scores (mean 12.04, 95% CI: 10.81–13.26) and greater CAC progression over five years (mean 23.03, 95% CI: 20.11–19.89) compared to inactive individuals (mean: 9.45, 95% CI: 8.76–10.14 and mean: 14.87, 95% CI: 13.18–16.55, respectively) [36] (Table 1). The authors propose that this increase in CAC might represent a stabilizing adaptation rather than elevated atherosclerotic risk. An editorial by Gulsin and Moss supports this perspective, suggesting that increased CAC in physically active individuals may indicate a protective response to chronic inflammation, promoting plaque stability [37] (Figure 1). They highlight that calcified plaques, as opposed to non-calcified plaques, are associated with a lower risk of rupture and subsequent cardiovascular events. This phenomenon, termed the “calcium paradox”, underscores the complexity of interpreting CAC scores. Gulsin and Moss have argued that, while CAC is a marker of coronary atherosclerosis, increased scores in the context of high PA should not be viewed solely as a negative outcome but may instead signify a shift toward a more stable plaque phenotype, emphasizing the overall cardiovascular benefits of PA.

### 3.2. Effects of High-Intensity and Prolonged Physical Activity on CAC Levels

Increased CAC scores are notably more common among individuals engaging in high-intensity or prolonged PA. Pavlovic et al. found that longer weekly PA duration was linked to higher CAC scores [46] (Table 2), while Kermott et al. observed increased CAC in individuals with the highest cardiorespiratory fitness levels [47] (Table 2). Long-term follow-up studies further support these findings. Aengevaeren et al. demonstrated that very vigorous exercise could accelerate CAC progression, with exercise intensity playing a larger role than volume [38] (Table 1). Additionally, Bhatia et al. also noted that higher PA is associated with increased CAC density [39] (Table 2). These findings highlight that, while regular PA is beneficial, particularly intense or prolonged PA may be associated with higher CAC scores. However, the stability and composition of these calcified plaques, rather than their mere presence, appear to be key to cardiovascular risk assessment.

### 3.3. Stabilizing Effects of Physical Activity on Plaque Composition

Research increasingly supports that calcified plaques in physically active individuals may exhibit greater stability than those in sedentary individuals, suggesting a potential stabilizing effect. Merghani et al. conducted a cross-sectional study of 152 masters athletes and age-matched controls, finding that male athletes had a higher prevalence of atherosclerotic plaques (44.3% vs. 22.2%). However, these plaques were predominantly calcified (72.7%), indicating a stable morphology [48] (Table 2). Similarly, Aengevaeren et al. studied lifelong exercise histories in 284 middle-aged men and observed that high lifetime PA volumes were associated with increased CAC. Those engaging in over 2000 MET-minutes per week showed significantly higher CAC scores (9.4 vs. 0.6–0.9) and prevalence of CAC (68%) [49] (Table 2). Nonetheless, these highly active individuals tended to exhibit plaques with a more benign composition, suggesting that, while high PA levels may increase CAC, they also enhance plaque stability, potentially reducing cardiovascular risks associated with softer, rupture-prone plaques.

### 3.4. Cardiovascular Benefits of Physical Activity Despite Increased CAC

Building on findings that high PA levels can elevate CAC, it is important to consider the broader cardiovascular benefits emphasized by both the ACC/AHA and ESC guidelines [10,54]. Regular PA is essential for cardiovascular health, as it reduces risks associated with high blood pressure, dyslipidemia, and insulin resistance. Exercise improves endothelial function, lowers systemic inflammation, and aids in weight management, all contributing to lower CVD incidence and mortality. The guidelines recommend a minimum of 150 min of moderate-intensity or 75 min of vigorous-intensity activity per week, with even greater benefits observed at higher levels of PA.

Supporting this perspective, the Cooper Center Longitudinal Study (CCLS) found that individuals with high PA levels are associated with prevalent CAC compared to less active individuals. However, at any given CAC level, higher PA was associated with lower mortality risk, suggesting a protective effect of exercise even with a large coronary calcification burden [55]. These results are consistent with the notion that, while higher levels of PA may be accompanied by greater CAC, they are also associated with lower cardiovascular risk. This evidence supports the safety and cardiovascular benefits of regular intensive PA, reinforcing its role in promoting heart health even in the presence of elevated CAC scores. 

## 4. Potential Mechanisms of PA-Induced CAC

Although the mechanisms underlying increased CAC with PA remain under investigation, several pathways beyond plaque stabilization have been proposed. High-intensity PA can cause significant hemodynamic changes, increasing shear stress on arterial walls, especially in regions of disturbed flow, like at arterial bifurcations. This shear stress may exacerbate endothelial injury, accelerating atherosclerosis in individuals with pre-existing plaques or endothelial dysfunction [56]. 

Another potential mechanism is oxidative stress. High-intensity PA increases reactive oxygen species (ROS) production in endothelial cells, which can contribute to vascular damage and the formation of atherosclerotic plaques through the oxidation of low-density lipoproteins (LDLs). While regular PA enhances antioxidant capacity by modulating the activity of pro- and antioxidant enzymes, transient ROS elevation during intense PA could contribute to increases in CAC [57]. 

Parathyroid hormone (PTH), a key regulator of calcium homeostasis, is also implicated. Patients with hyperparathyroidism demonstrate higher rates of CVD and vascular calcifications. Exercise-induced increases in PTH levels suggest a potential mechanism for exercise-related atherosclerosis, with repeated exposure to elevated PTH post-exercise potentially accelerating coronary atherosclerosis [58]. 

Inflammatory processes also play a key role in the development and progression of atherosclerotic plaques. Oxidized LDL can recruit immune cells and release proinflammatory cytokines, promoting plaque growth [59]. While regular exercise has an anti-inflammatory effect, acute high-intensity exercise can provoke a transient inflammatory response that may accelerate atherosclerosis in individuals engaging in frequent, prolonged, intense exercise regimens.

## 5. Contradictory Findings and Protective Effects of Exercise

Despite evidence suggesting that high PA levels can increase CAC, other studies have found an inverse or non-significant relationship between PA and CAC progression. Shuval et al. investigated the relationship between high-volume leisure-time aerobic PA and CAC progression in 8771 men and women and found a cross-sectional association between higher PA and higher CAC in men, although there was no significant longitudinal association between high PA with CAC progression [42] (Table 1). However, their study had methodological concerns, such as violations of the proportional hazards assumption in their Cox regression model, which requires constant hazard ratios for covariates over time. High initial levels of CAC among individuals who exercised extensively did not further increase with continued exercise, indicating a time-varying effect of PA on CAC progression. This could lead to potentially inaccurate hazard ratios. Additionally, transitioning from electron beam tomography to a 64-slice scanner during their study might have introduced variability in CAC measurements, influencing their results. These issues suggest that their conclusions may not fully capture the temporal dynamics of the relationship between PA and CAC.

Several additional studies suggest that moderate to vigorous physical activity may reduce CAC. Kamimura et al. reported that ideal PA levels are associated with lower high-sensitivity C-reactive protein levels, a lower prevalence of CAC, and reduced coronary heart disease (CHD) incidence [51] (Table 2). Jae et al. found that higher fitness levels correlate with a lower prevalence of subclinical atherosclerosis, including CAC, especially in men with cardiometabolic syndrome [52] (Table 2). Similarly, Kwaśniewska et al. demonstrated that maintaining high levels of PA in middle and older age may protect against atherosclerosis as measured by CAC, intima–media thickness (IMT), and reactive hyperemia index (RHI) [44] (Table 1). Gabriel et al. highlighted that higher levels of moderate- to vigorous-intensity physical activity (MVPA) are associated with lower subclinical disease markers in older women [45] (Table 1). Furthermore, a long-term follow-up study by Chedid et al. found that higher METs are linked to lower CAC scores [43] (Table 1). These studies illustrate that the relationship between PA and CAC is complex and influenced by factors such as population demographics, measurement methods, and types and intensities of PA. While high levels of PA may increase CAC in some individuals, the protective cardiovascular effects of exercise—such as reduced inflammation, improved endothelial function, and the control of traditional risk factors—should not be overlooked.

## 6. Future Research Directions

The inconsistent and even conflicting results pertaining to the association between PA and CAC underscore the need for further research to provide more conclusive insights. Future studies should incorporate objective measures of PA, such as wearable fitness trackers, to yield more accurate data. Wearable devices can collect real-time information on the frequency, intensity, and duration of PA, which may provide a more comprehensive and accurate measurement of activity levels than that provided by self-reports. This approach addresses the limitations of self-reported data, which are often affected by recall bias and inaccuracies.

Additionally, assessing calcium volume and density, rather than relying exclusively on the Agatston score, will provide a clearer understanding of calcification and its relationship with exercise. The Agatston score measures the amount of calcification without providing information on specific types of calcified plaques. By measuring calcium volume and density, researchers can distinguish between stable, dense calcifications and less stable, softer plaques. This differentiation is critical, as stable calcified plaques are less prone to rupture and subsequent cardiovascular events. Thus, a detailed analysis of calcium characteristics can provide a clearer perspective on how increased CAC in physically active individuals impacts plaque stability and progression.

Longitudinal studies are essential for establishing causality and clarifying the long-term effects of various types and intensities of PA on CAC progression. Population-specific research is needed to account for differences in age, gender, race, and genetic background, which can guide personalized exercise recommendations. Future investigations should also compare the effects of different exercise intensities and durations to identify the optimal balance that maximizes health benefits while minimizing adverse effects. The use of a consistent methodology across studies—including standardized measurement techniques, study designs, and data analysis methods—is essential to reconcile conflicting findings. Addressing these research priorities will clarify the relationship between exercise and CAC and facilitate the optimization of cardiovascular benefits.

## 7. Conclusions

This review underscored the paradoxical relationship between PA and CAC. Although PA is broadly recognized for its cardiovascular benefits, emerging evidence suggests that high levels of PA, especially vigorous exercise, might be associated with elevated CAC scores. Key studies have observed that individuals with high levels of PA often have higher CAC scores than those with lower activity levels. This increase in CAC score in highly active individuals necessitates a balanced interpretation. Elevated CAC scores do not inherently indicate a greater risk of cardiovascular events if calcified plaques are stable. Clinicians should consider the overall cardiovascular health benefits of PA when interpreting CAC scores. While continued PA should be promoted, it should be accompanied by personalized clinical assessments to consider individual risk factors and health conditions.

## Figures and Tables

**Table 1:** Longitudinal studies on the impact of PA on CAC.

Longitudinal Study	Study Population	PA Measurement Method	CAC Distribution	Impact of PA on CAC
Aengevaeren et al. (2023) [38]	291 men from the MARC-1 cohort (92% of total), baseline age 54 years (range: 50–60) with a 6-year follow-up. Of these, 287 were included for CAC analysis and 284 for plaque analysis.	PA characteristics (type, duration, frequency, performance level) were assessed at two time points (MARC-1 and MARC-2) using a validated questionnaire. PA volume categorized into tertiles (<1000, 1000–2000, >2000 MET-min/week), with intensity classified as moderate (3–6 METs), vigorous (6–9 METs), or very vigorous (≥9 METs).	- Baseline CAC: median 1 [IQR: 0, 32]. - Follow-up CAC: median 31 [IQR: 10, 132]. - CAC ≥ 100 increased from 15% to 31% over time.	- Vigorous exercise was negatively associated with CAC progression (β −0.05 per 10%, *p* = 0.02). - Very vigorous exercise was positively associated with CAC progression (β 0.05 per 10%, *p* = 0.01).
Bhatia et al. (2022) [39]	Among 906 South Asian participants (mean age 52.4 years), 701 completed both baseline (Exam 1: 2010–2013) and follow-up assessments (Exam 2: 2015–2018). After exclusions, data from 387 individuals were analyzed (mean age 58.5 ± 9.0 years at Exam 1 and 62.8 ± 8.9 years at Exam 2).	Physical activity was assessed through the Typical Week’s Physical Activity Questionnaire.	- Overall CAC volume at Exam 1: 36 mm^3^ [IQR: 0, 167]; at Exam 2: 105 mm^3^ [IQR: 27, 360]. - Peak CAC density at Exam 1: 307 [IQR: 0, 453]; at Exam 2: 482 (IQR: 360, 622).	- Higher physical activity was linked to increased CAC density in women (LCx), notably, and men (LM), with minimal volume change. - Exercise level (MET-min/week) was positively associated with CAC density progression (β = 0.19 per 10 MET-min/week, *p* = 0.01), with a smaller effect on CAC volume.
Sung et al. (2021) [36]	25,485 participants (22,741 men, 2744 women; mean age, 42.0 ± 6.1 years) with two or more CAC measurements completed the IPAQ-SF between 2011 and 2017.	Physical activity was measured using the Korean IPAQ-SF, categorizing participants as inactive, moderately active (≥600 MET-min/week), or HEPA (≥1500 MET-min/week for vigorous or ≥3000 MET-min/week for combined activities).	- Overall mean CAC: 14.7 (±75.7) AU. - Baseline mean CAC: 9.45 (inactive), 10.20 (moderately active), 12.04 (HEPA). - 5-year mean CAC: 24.32 (inactive), 28.27 (moderately active), 35.06 (HEPA).	- Higher PA was associated with greater CAC at baseline. - Over 5 years, CAC increased by 3.20 in moderately active and 8.16 in HEPA participants compared to that of inactive participants. - For CAC = 0 participants, the risk of CAC > 0 was higher in the HEPA group (HR 1.21, 95% CI 1.05–1.38).
Laddu et al. (2017) [35]	3175 participants (mean age, 25.4 ± 0.5 years; 47.4% Black, 56.6% women) from the CARDIA study, recruited between 1985 and 1986 from 4 U.S. cities.	Self-reported PA was measured using the CARDIA Physical Activity History questionnaire. A total of 13 activities were categorized as vigorous or moderate. Participants were grouped as below PA guidelines (57.1%), meeting guidelines (34.5%), and exceeding guidelines by three times (8.4%).	CAC ≥ 100 AU was observed in 16.6% of participants below PA guidelines, 21.4% meeting PA guidelines, and 11.9% exceeding guidelines by three times.	- Participants exceeding the PA guidelines by three times had the highest prevalence of CAC > 0 (41.8%) and CAC > 20 (25.0%) at year 25 (*p* < 0.001). - White men exceeding PA guidelines had higher odds of CAC > 0 (AOR 1.86; 95% CI: 1.16, 2.98), with no significant associations for Black men, Black women, or White women.
Shah et al. (2016) [40]	- 4872 young adults (mean age of 24.8 years, 54.5% female) from the CARDIA study, recruited in 1985–1986 from four U.S. cities. - Year 7 analysis included 2472 participants for CRF analysis.	Cardiorespiratory fitness (CRF) was assessed using a graded, symptom-limited maximal exercise test with the modified Balke treadmill protocol, with a second test at year 7 to evaluate changes in CRF.	CAC prevalence at year 25 by treadmill duration: tertile 1, 24.3%; tertile 2, 25.9%; tertile 3, 34.6%.	No significant association was found between baseline or changes in CRF and CAC presence at years 15, 20, or 25, even after adjusting for age, race, sex, obesity, and cardiovascular risk factors. Exercise test duration and 7-year CRF changes did not predict CAC presence at follow-up.
Hamer et al. (2012) [41]	443 participants (mean age 66 ± 6 years, range 57–79) from the Whitehall II cohort (2009/2010).	Physical activity measured by accelerometers over seven days, categorized by intensity (sedentary, light, MVPA). Self-reported physical activity data from 1997 to 2004 were also analyzed.	Coronary calcium scores ranged from 0 to 3510 (median: 10.8; SD: 364.7), with 63.9% of participants (n = 283) having detectable CAC.	No significant association was found between PA levels and CAC presence. Average MVPA did not differ across CAC categories (*p*-trend = 0.72). Inverse relationships between MVPA and CAC were nonsignificant after age adjustment. Longitudinal self-reported PA from 1997 to 2004 showed no association with CAC in 2009/2010.
Shuval et al. (2024) [42]	The study included 8771 healthy adults aged 40 and older (mean age: 50.2 ± 7.3 years for men; 51.1 ± 7.3 years for women) who visited the Cooper Clinic in Dallas, Texas, from 1998 to 2019, with an average follow-up of 7.8 years.	PA was reported at baseline and follow-up, assessed both continuously per 500 MET-minutes per week and categorically as <1500, 1500–2999, or ≥3000 MET-min/week.	- In men, the mean CAC score was 95.9 (SD: 301.0) at baseline and 222.1 (SD: 485.9) at follow-up (*p* < 0.001). - In women, the mean CAC score was 20.0 (SD: 93.1) at baseline and 48.0 (SD: 158.1) at follow-up (*p* < 0.001).	- The mean annual CAC progression rate from baseline was 28.5% in men and 32.1% in women, independent of mean PA. The difference in CAC progression was 0.0% per 500 MET-min/week for both genders (95% CI for men: −0.1% to 0.1%; 95% CI for women: −0.4% to 0.5%). - Baseline PA was not associated with CAC progression to a clinically significant threshold of 100 AU or more during follow-up.
Chedid et al. (2023) [43]	241 participants (203 men, 38 women) with stable CAD; mean age: 62.8 ± 7.8 years for men and 64.1 ± 6.7 years for women.	Maximal exercise treadmill testing at baseline with METs calculated to assess cardiorespiratory fitness.	Baseline CAC was lower in women than men: median [IQR]: 106.7 [25.3, 294.1] AU vs. 535.3 [182.9, 1368.9] AU, *p* < 0.001.	In women, PA significantly predicted CAC: each 1 MET increase corresponded to a 77-unit lower CAC at baseline and 76-unit lower CAC at 30 months. In men, age was the strongest predictor; METs did not predict CAC.
Kwaśniewska et al. (2014) [44]	101 men (aged 50–77 years; mean age: 59.7 ± 9.0) from the Healthy Men Clinic at the Medical University of Lodz (1985–2012).	PA data were collected via interviews (1985–2002). Exercise-related energy expenditure (EE) was calculated weekly in kcal/week. From 2003, PA was assessed using the Seven-Day PA Recall Questionnaire. Participants were categorized into tertiles based on EE: low-to-moderate (<2050 kcal/week), high (2050–3840 kcal/week), and very high (>3840 kcal/week).	Mean/median CAC scores for PA groups: - Low-to-moderate PA: 286.1 ± 361.9 (median: 121.3); - High PA: 10.7 ± 28.9 (median: 1.7); - Very high PA: 106.1 ± 278.3 (median: 6.30).	- Men with high PA had the lowest CAC scores, while CAC > 400 was more common in the low-to-moderate and very high PA groups. - Half of the men with high or very high PA had a CAC score of 0, compared to only 12.1% in the low-to-moderate group.
Gabriel et al. (2013) [45]	148 women had valid accelerometer and CAC data, with mean ages of 61.9 ± 1.7 years at EBT1 and 73.2 ± 1.7 years at EBT4.	PA data were collected through self-reported questionnaires and accelerometers at EBT4.	No detectable CAC (n = 37, 25%), incident CAC (n = 46, 31.1%), prevalent CAC (n = 65, 43.9%)	At EBT4, the prevalent CAC group had significantly lower levels of MVPA and sustained MVPA time compared to the no detectable CAC group.

**Table 2:** Cross-sectional studies on the impact of PA on CAC.

Cross-Sectional Study	Study Population	PA Measurement Method	CAC Distribution	Impact of PA on CAC
Pavlovic et al. (2024) [46]	23,383 men (mean age 51.7 ± 8.3 years) from the Cooper Center Longitudinal Study who underwent preventive exams, completed PA questionnaires, and received CAC scans between 1998 and 2019.	Participants reported weekly leisure time PA over three months using a questionnaire. PA was grouped by intensity (1, 3–5.9, 6–8.9, 9–12 METs) and weekly duration (0, >0-<2, 2–<5, ≥5 h).	- Mean CAC: 174.8 AU (SD: 543.6); 23.5% had CAC ≥ 100 AU. - Men in the 9–12 METs group had a significantly lower mean CAC (76 ± 296.7 AU) than the inactive group (226.9 ± 625.9 AU). - The unadjusted mean CAC was lowest in the >0–<2 h group (143.3 AU) and highest in the inactive group (226.9 AU).	- A higher PA intensity was linked to a lower CAC. - A longer PA duration was associated with a higher CAC. - The risk of CAC ≥ 100 AU decreased with a higher PA intensity but increased with a longer weekly duration.
Kermott et al. (2019) [47]	2946 men (mean age 51.7 ± 7.5 years) from the Mayo Clinic Executive Health Program who underwent CAC assessments and treadmill testing between 1995 and 2008.	Exercise workload was measured in METs and quantified as functional aerobic capacity (FAC) from treadmill tests. FAC was categorized into four groups: A (≤69%), B (70–99%), C (100–129%), and D (≥130%).	- Median CAC (IQR) without family history of premature CAD: 21 (0–88), 3 (0–59), 1.4 (0–54), and 13 (0–100) for FAC ≤ 69%, 70–99%, 100–129%, and ≥130%. - Median CAC (IQR) with a family history of premature CAD: 10 (0–158), 6 (0–66), 6 (0–77), and 34 (0–270) for the same FAC groups.	- CAC was significantly higher in patients with FAC ≥ 130%, regardless of family history. - Adjusted for age, BMI, and family history, FACs of 70–99% and 100–129% were associated with a lower CAC compared to FAC ≥ 130%.
Merghani et al. (2017) [48]	- Masters athletes (mean age of 54.4 ± 8.5 years; 70% men; 92% White) recruited from elite UK running and cycling clubs. - Control group: Healthy individuals recruited from London hospitals, matched for age, sex, and Framingham 10-year CAD risk.	Masters athletes were defined by their endurance activity: running ≥ 10 mi/week or cycling ≥ 30 mi/week for ≥10 years, with ≥10 endurance events over 10 years.	- Median CAC score was 0 for both athletes and controls. - In male athletes with CAC ≥ 1, the median CAC was significantly higher than in sedentary males with CAC ≥ 1 (86 vs. 3, *p* = 0.02).	- Male athletes had a higher prevalence of CAC ≥ 300 AU (11.3%) compared to sedentary males (0%, *p* = 0.009).
Aengevaeren et al. (2017) [49]	284 men (mean age 55.0 ± 6.5 years, 100% White) who engaged in competitive or recreational sports.	Participants reported their lifelong exercise history. MET scores were calculated using the Compendium of Physical Activities, with exercise volume categorized as <1000, 1000–2000, or >2000 MET-min/week.	- Median CAC score: 35.8 [9.3–145.8]. - CAC by exercise volume: >2000 MET-min/week group: 9.4 [0–60.9], <1000 MET-min/week group: 0 [0–43.5].	- Higher exercise volumes were linked to increased CAC prevalence. - Athletes with >2000 MET-min/week had a higher CAC prevalence (68%) than those with <1000 MET-min/week (43%). - The highest exercise volumes were associated with more calcified, compositionally benign plaques.
DeFina et al. (2014) [50]	5341 women, aged 40–90 years (mean age: 52 years), who underwent treadmill testing and CAC scanning (1997–2007).	Fitness was assessed using the modified Balke treadmill protocol and converted to METs. Participants were divided into age-specific fitness quintiles and categorized as “unfit” (quintile 1), “moderately fit” (quintiles 2–3), and “highly fit” (quintiles 4–5).	Overall, 19.9% had detectable CAC, and 6.8% had CAC ≥ 100.	- Higher fitness levels were significantly associated with lower CAC scores, but not for CAC ≥ 100. - After adjusting for cardiovascular risk factors, no significant association remained between fitness levels and CAC at any threshold.
Kamimura et al. (2021) [51]	Of the 5306 original Jackson Heart Study participants, 2420 (mean age: 54 ± 13) were included in the CAC analysis.	PA was self-reported and categorized into American Heart Association (AHA) levels: ideal (≥150 min/week of moderate or ≥75 min/week of vigorous), intermediate (1–149 min/week of moderate or 1–74 min/week of vigorous), and poor (0 min/week).	The mean CAC score for the cohort was 127 ± 372. By PA levels, the mean CAC scores were 161 ± 436 for the poor PA group, 99 ± 301 for the intermediate PA group, and 99 ± 311 for the ideal PA group.	Ideal PA was associated with a lower prevalence of CAC Agatston score ≥ 100 (OR: 0.70; 95% CI: 0.51–0.96) compared to poor PA.
Jae et al. (2016) [52]	2107 men who participated in a health screening program that included CAC and CIMT measurements.	Fitness was directly measured using peak oxygen consumption during cardiopulmonary exercise testing to exhaustion.	Participants without cardiometabolic syndrome had a median CAC score of 2 (IQR: 0–52) and those with cardiometabolic syndrome had a median CAC score of 6 (IQR: 0–73).	- Higher fitness levels were associated with a lower prevalence of CAC (OR: 0.69; 95% CI: 0.55–0.88). - Fit participants with CMS showed similar odds for CAC prevalence (OR: 1.12; 95% CI: 0.85–1.47) as those without CMS.
Storti et al. (2010) [53]	173 younger postmenopausal (PM) women (mean age: 56.8 ± 2.9 years) from the WOMAN study and 121 older PM women (mean age 73.9 ± 3.8 years) from the Walking Women Follow-up (WWF) study, all with complete PA and CAC data.	PA was objectively measured using a pedometer over a 7-day period in both cohorts.	Detectable CAC was found in 57% of WOMAN participants and 74% of WWF participants. The median CAC score was 1.4 (0–23.3) for WOMAN participants and 38.8 (0–264.4) for WWF participants.	Among the WWF participants, a statistically significant inverse association was observed between pedometer steps and CAC (*p*-trend = 0.002). No association was found in the WOMAN study’s participants.

## Data Availability

Not applicable.
